# Quantitative bead–based multiplex assay for simultaneous determination of IgG concentrations of pertussis toxin, filamentous hemagglutinin, pertactin, diphtheria, tetanus, Haemophilus influenzae b, and hepatitis B in human serum samples

**DOI:** 10.3389/fimmu.2025.1587567

**Published:** 2025-07-01

**Authors:** Vishal Rathod, Sagar Katke, Sumant Patil, Sachin Bhandare, Laxmikant Kadam, Manish Gautam, Prabhu Gumma, Krishna Manoj Kumar, Laura Hassall, Cathy Asokanathan, Alex Douglas-Bardsley, Kevin Markey, Sumit Gupta, Harish Rao, Sameer Parekh, Pramod Pujari, Hitt Sharma, Umesh Shaligram, Sunil Gairola

**Affiliations:** ^1^ Clinical Bioanalytical Laboratory, Serum Institute of India Pvt Ltd., Pune, Maharashtra, India; ^2^ Science and Research, Medicines, and Healthcare Products Regulatory Agency, South Mimms, United Kingdom

**Keywords:** ELISA, luminex, multiplex immunoassay, combination vaccines, Haemophilus influenzae B, Pertussis, Hepatitis B

## Abstract

**Background:**

Multiplex serological assays provide opportunities for seroprevalence studies and for evaluating antibodies post-vaccination. In this report, we describe the development and validation of a seven-plex bead-based assay for quantifying human immunoglobulin G (IgG) antibodies against pertussis toxin (PT), filamentous hemagglutinin (FHA), pertactin (PRN), diphtheria toxoid (DT), tetanus toxoid (TT), Haemophilus influenzae b (Hib), and hepatitis B (Hep B) using international reference standards.

**Methods:**

Existing international human reference sera standards are tailored for monoplex assays and, therefore, require characterization for multiplex assays. The reference standards for pertussis (06/142), diphtheria (10/262), tetanus (13/240), Hib (09/222), and Hep B (07/164) were characterized for their suitability in the assay. The purified antigens (PT, FHA, PRN, DT, TT, Hib, and Hep B) were coupled to spectrally unique magnetic carboxylated beads. The method was validated according to the United States Food and Drug Administration (US FDA), European Medicines Agency (EMA), and International Council for Harmonization Multidisciplinary (ICH M10) guidelines. Validation parameters, such as precision, accuracy, dilution linearity, assay range, robustness, and solution stability, were assessed.

**Results:**

An equi-mix of an international reference standard for Hep B (07/164) and Hib (09/222) provided the best dynamic range for the seven-plex assay. Method validation was conducted using a panel of human serum samples that included samples from vaccinated healthy volunteers, non-vaccinated volunteers, negative controls, and international reference standards. Assay specificity using inhibition experiments demonstrated specificities of 98%, 95%, 93%, 98%, 97%, 97%, and 98% for DT, TT, FHA, PRN, PT, Hib, and Hep-B, respectively. Spike recoveries of 80%–120% were demonstrated in different matrices, including those of hemolytic and lipemic sera samples. The precision and accuracy were confirmed by evaluating a panel of human serum samples obtained from vaccinated individuals. The assay demonstrated coefficients of variation (CV) of ≤ 20% across all assays, regardless of run, day, or analyst. This method demonstrated strong agreement with conventional commercially available assays, highlighting the advantages of multiplexing over traditional enzyme-linked immunosorbent assays (ELISAs).

## 1 Introduction

Combination vaccines undoubtedly provide numerous public health benefits, including improved vaccine access and immunization coverage (1). Paediatric combination vaccines began with the combination of individual diphtheria (D), tetanus (T), and pertussis (DTP) vaccines into a single product, which became the cornerstone of the immunization program. Subsequently, additional antigens were incorporated into the combinations, enabling the integration of diphtheria toxoid (DT), tetanus toxoid (TT), and acellular pertussis antigens (DTaP) with other routine vaccines such as inactivated polio vaccine (IPV), Haemophilus influenzae type b vaccine (Hib), and hepatitis B vaccine (Hep B) antigens (2).

Immunogenicity testing for DT- and TT-based combinations is carried out through serology (3, 4). Typically, commercially available kits or conventional enzyme-linked immunosorbent assays (ELISA) are used for immunogenicity testing of vaccines (5). However, most of these kits are not designed for immunogenicity assessment and thus require detailed validation. Additionally, commercial kits or conventional ELISA detect one analyte (monoplex), thus requiring a large volume of sera for assaying combination vaccines, which poses a challenge for paediatric vaccines (6, 7). Multiplex assay platforms that can quantify antibodies against multiple antigens are efficient for the concurrent analysis of immunoglobulin G (IgG) concentrations against combination vaccine antigens (8, 9). Several studies have previously described the accuracy and high-throughput advantage of the Multiple Analyte Profiling Technology (x-MAP^®^; Luminex Corp., Austin, TX) platforms as an alternative approach for evaluating vaccine-elicited binding antibodies (10–15). Previously, we reported the development and validation of a five-plex bead-based assay targeting DT, TT, pertussis toxin (PT), filamentous haemagglutinin (FHA), and pertactin (PRN) antigens (16). Given that many TT- and DT-based vaccines incorporate additional antigens, such as Hib and Hep B, it would be exceptionally beneficial to expand the existing five-plex or existing multiplex assays by including antigens such as Hib and Hep B antigens.

Traditionally, the quantification of IgG concentrations against both Hib and Hep B has been performed using commercial ELISA kits (17, 18). The Hib antigen is based on a repeating unit known as poly-ribosyl-ribitol phosphate (PRP) (19), and the immune response is evaluated by measuring the serum anti-PRP antibodies. Protection from Hib disease is strongly associated with the presence of anti-PRP antibodies. The minimum levels of anti-PRP antibodies considered to provide short-term and long-term protection against Hib disease are 0.15 µg/ml and 1 µg/ml, respectively (20). For Hep B antigens, various commercial assays have been used to assess the efficacy of vaccines. Currently, anti-Hep B antibody levels are expressed in international units (IU), and concentrations >10 mIU/ml are considered protective (21). There are limited or no reports on multiplex assays that combine Hib and Hep B antigens with TT, DT, or aP antigens or any other antigens. This study is an attempt in this direction.

Immunogenicity assays play a pivotal role in the vaccine licensing process, and they must undergo thorough validation. Current regulatory guidelines stress the use of international standards to facilitate the comparison of results across different trials (22–24). While international reference standards exist for vaccine antigens, they are more tailored for monoplex assays (25). With the industry moving toward multiplex assays, it is imperative to adapt existing international reference standards to effectively validate and standardize multiplex assays. The aims of this study are (a) the development and validation of the multiplex assay and (b) the characterization and suitability assessment of international reference standards for pertussis (06/142), diphtheria (10/262), tetanus (13/240), Hib (09/222), and Hep B (07/164) for their suitability in the multiplex serology assay.

## 2 Materials and methods

### 2.1 Antigens, reagents, and ELISA kits

Purified PT, FHA, PRN pertussis antigens, DT, TT, Hib, and Hep B antigens were sourced from Serum Institute of India Pvt. Ltd. (SIIPL, India). All antigens were tested for content and purity. The protein content of the antigens was estimated using a validated bicinchoninic acid (BCA) assay (26). Purity was tested using a validated SDS-PAGE assay. According to the manufacturer’s recommendation, antigens were stored in aliquots at −20°C or lower temperatures. R-phycoerythrin (R-PE)–conjugated to anti-human antibody was obtained from Southern Biotech, United States of America. Beads (carboxylated microspheres) were procured from Luminex Corporation, USA, and 1-ethyl-3-(3-dimethyl aminopropyl) carbodiimide (EDAC) was obtained from Bio-Rad Laboratories, India. Sulfo-N hydroxysulfosuccinimide (sulfo-NHS) was procured from Thermo Fisher Scientific, USA. Bovine serum albumin (BSA) was obtained from Sigma-Aldrich, India. Tween 20 was purchased from SD Fine Chem Limited, India. The commercial ELISA kits for quantification of IgG against PT and FHA antigens were procured from Euroimmun, Germany. Kits for PRN, DT, TT, Hib, and Hep B antigens were procured from IBL, USA, and Bio-Rad India, respectively.

### 2.2 World Health Organization international standards and reference reagents

World Health Organization (WHO) International Standards (IS) and reference reagents (RR) were purchased from the National Institute for Biological Standards and Controls (NIBSC), United Kingdom. A total of six WHO reference standards were used in the study: 06/142, 10/262, TE-3, 13/240, 07/164, and 09/222. The unitages of the reference standards are reported in the international unit IU/ml and µg/ml, traceable to the international reference standard. The WHO reference reagent for Pertussis Antiserum Human (06/142) is a freeze-dried preparation of pooled re-calcified human serum with an assigned anti-PT IgG content of 106 IU/ampoule, an anti-FHA IgG content of 122 IU/ampoule, and an anti-69K IgG content of 39 IU/ampoule. WHO IS for Diphtheria Antitoxin Human (10/262) is a freeze-dried preparation of normal human IgG with a diphtheria antitoxin potency of 2 IU/ampoule. For tetanus, the 1st WHO International Standard for Anti-Tetanus Immunoglobulin Human (TE-3) is a freeze-dried preparation of human tetanus immunoglobulin with an assigned unitage of 120 IU/ampoule. 13/240 is the 2nd WHO international standard, with an assigned unitage of 45 IU/ampoule. 09/222 is CE-marked human anti-Haemophilus influenzae b (09/222) reference sera with an assigned IgG content of 34.7 µg/ampoule. 07/164 is the 2nd WHO international standard for anti-hepatitis B surface antigen immunoglobulin with an assigned unitage of 100 IU/ampoule. All reference standards were handled as per the manufacturer’s recommendations. The unitages for these standards per the NIBSC instructions for use are also provided in **Table 1**.

**Table 1:** WHO reference standards with respective unitages.

Standards	Batch no.	Antibodies (IgG)	Unitages
Pertussis Antiserum Human	06/142	PT	106 IU/ampoule
		FHA	122 IU/ampoule
		Anti-69K (Pertactin)	39 IU/ampoule
Diphtheria Antitoxin Human	10/262	DT	2 IU/ampoule
1st International Standard for Tetanus Immunoglobulin, Human	TE-3	TT	120 IU/ampoule
2nd International Standard -Tetanus Immunoglobulin Human	13/240	TT	45 IU/ampoule
Human anti-Haemophilus influenza b reference serum	09/222	Hib	34.7 µg/ampoule
Anti-hepatitis B surface antigen (anti-HBs) immunoglobulin, human	07/164	Hep B	100 IU/ampoule

IgG, Immunoglobulin G; PT, Pertussis toxin; FHA, Filamentous hemagglutinin; Anti-69K, Pertactin; DT, Diphtheria toxoid; TT, Tetanus toxoid; Hib, Haemophilus influenza b; Hep B, Hepatitis B; IU, International units; WHO, World Health Organization; µg, microgram.

### 2.3 Coupling of antigens to carboxylated microspheres

Antigens (PT, FHA, PRN, DT, and TT) were coupled to the spectrally unique magnetic carboxylated microspheres using previously reported procedures based on EDAC chemistry (16). For Hib and Hep B, coupling procedures were optimized: microspheres were activated with a carbodiimide derivative, EDAC hydrochloride-containing buffered solution. The intermediate carboxyl groups formed on the beads by reaction with EDAC were stabilized using a sulfo-NHS solution. This was followed by 3× washing steps using a magnetic separator. Respective antigens of varying concentrations (1, 5, and 10 µg) were added to the activated beads and kept in the dark for 2h under constant mixing at 15–30 revolutions per minute (rpm). The resulting mixture was washed, and the supernatant was discarded during every washing step. After three stages of pelleting and washing, coupled beads were blocked using 1% BSA in 1× PBS buffer for 30 min and kept in a storage buffer until further use (0.1% w/v BSA in PBS containing 0.05% sodium azide and 0.02% Tween 20).

### 2.4 Development of multiplex reference standard for seven-plex assay

**Figure 1:**
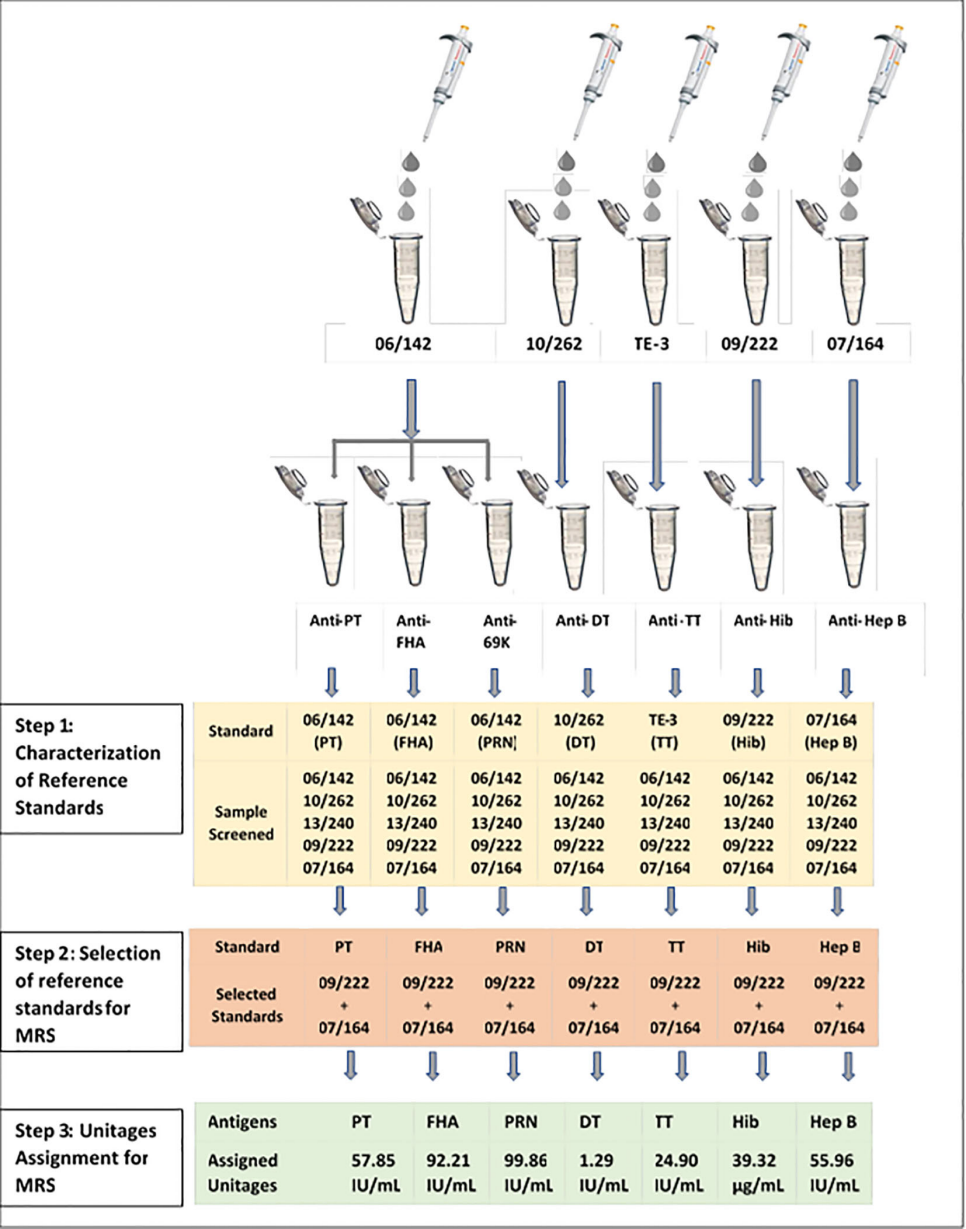
Development of multiplex reference standard (MRS) using international reference standards: PT, pertussis toxin; FHA, filamentous haemagglutinin; PRN, pertactin; DT, diphtheria toxoid; TT, tetanus toxoid; Hib, Haemophilus influenza b; Hep B, hepatitis; MRS, multiplex reference standard; IU/ml, international units per milliliter, µg/ml, microgram per milliliter.

The multiplex immunoassay (MIA) requires a reference standard that should be positive for antibodies (IgG) against all seven antigens. International standards currently available for these antigens are designed for the calibration of monoplex ELISAs. Previously, we reported the characterization and use of international reference standards for use in multiplex assays (16). A similar approach was used for the development of a reference standard against seven antigens. Briefly, all reference standards were screened for the antibodies against all seven antigens. Monoplex bead-based assays were carried out for screening and determination of antibody concentration in available reference sera 06/142, 10/262, 13/240, 09/222, and 07/164 against each antigen as described in **Figure 1**. Monoplex assay here refers to a setup wherein only one bead per target antigen is added instead of seven different beads. Twofold serial dilutions of the respective human reference standards and samples were performed from 1:100 to 1:12,800 and were added to the monovalent beads. Assay blanks were included in the plate as a control. All the incubation conditions, the number of washes, buffers, and instrument settings were similar to the multiplex assay procedure (Section 2.5).

The antibodies against PT, FHA, and PRN in each of these standards were quantified using 06/142 as the reference standard. The antibodies against TT, DT, Hib, and Hep B were quantified using TE-3, 10/262, 09/222, and 07/164 reference standards, respectively. A total of 6 runs were carried out to quantify IgG against TT, DT, PT, FHA, PRN, Hep B, and Hib reference standards. Based on the antibody concentrations for all seven antigens, an equi-mix (1:1) of two reference standards, 09/222 and 07/164, was selected for preparation of the Multiplex Reference Standard. **Figure 1** provides the schematic presentation of the approach used to develop the MRS using international reference standards. Reference standard development for MIA followed WHO recommendations on developing secondary reference standards (27).

### 2.5 Heptaplex immunoassay

The MRS, an equi-mixture of 09/222 and 07/164, was prepared as detailed in section 2.4 and used as the reference standard for the heptaplex assay. The assay involved preparation of serial dilutions of MRS and test sera samples in Luminex assay buffer (LAB). MRS is diluted from 1:200 to 1:25,600 to prepare eight serial dilutions covering the assay range for all seven antigens. Test sera samples are assayed at a minimum dilution of 1:100. Bead mixture (containing antigen-coupled magnetic beads for seven antigens) is prepared in LAB to yield a minimum of ~4,000 beads per region in 50 µl volume. Bead mixture is then added (50 µl/well) to a 96-well filter plate, followed by a 1× wash step using LAB. The next step involves the addition of 50 µl of MRS and test sera samples to beads in a 96-well filter plate. This is followed by incubation of the filter plate in the dark at 37°C for 1h with shaking at 150 rpm. Post incubation, the plate is washed (*n* = 3) with LAB, and 50 µl of 1:100 diluted goat anti-human antibody conjugated to R-phycoerythrin (R-PE) is added to all the wells of the plate. The plates are incubated again in the dark at 37°C for 30 min with shaking at 150 rpm, followed by a washing step (*N* = 3) with LAB. In the post-washing step, 100 µl of LAB is added to each well, followed by reading of the plate in the Protein Suspension Array System (Bioplex-200). Assay system suitability criteria for reference and sample as outlined in section 2.10.1 are monitored for validity of results.

### 2.6 Verification of assigned MRS unitages using international reference standards

The unitages of the MRS were verified by running the international reference standards as controls using the heptaplex assay as detailed in section 2.5. The unitages obtained for reference standards 06/142, 10/262, 13/240, 09/222, and 07/164 against MRS were compared with their designated unitages. An acceptance criterion of 80%–120% concordance with their designated unitages was followed as a criterion for successful verification.

### 2.7 Internal quality controls

Internal quality controls (IQCs) stock standards were prepared by mixing equal volumes (1:1:1) of 09/222, 07/164, and 13/240. The stock standard unitages were determined against the MRS as PT (101.22 IU/ml), FHA (163.98 IU/ml), PRN (131.68 IU/ml), DT (3.11 IU/ml), TT (30.84 IU/ml), Hib (31.24 µg/ml), and Hep B (39.94 IU/ml). Using stock standard, three different IQC levels (IQC-1 to IQC-3) were prepared using LAB. Acceptance limits were established by repeated testing of the IQCs (*n* = 15). Acceptable ranges for the estimates were set as mean ± 2 standard deviations (SDs) of the IgG concentrations to each antigen.

### 2.8 Human serum samples for method validation

Serum samples (vaccinated and unvaccinated) used for method validation were collected from volunteers aged >18 years working at SIIPL, India, after obtaining informed consent. The selected sera samples (*n* = 15) for the study are presented in **Table 2**. The selected sera samples covered various concentrations, including negative, low, medium, and high concentration samples. All serum samples were used as per the local regulations and guidelines and approved by the Independent Research Ethics Committee, Pune (IEC No. IRECP/006/2022). The sera samples were tested using MIA to quantify antibodies against all seven antigens. Based on concentrations, six different panels were designed for method validation. Panel 1: Vaccinated samples for precision and accuracy containing high, medium, and low IgG concentrations; Panel 2: Non-vaccinated or negative samples for selectivity; Panel 3: Matrix representing hemolytic and lipemic samples for selectivity; Panel 4: Antibody-depleted human sera (ADHS) for selectivity; Panel 5: High titer sera for dilution linearity; Panel 6: MRS and IQCs for solution stability.

**Table 2:** Sera panel used for assay validation.

Sample no.	Panel 1	Panel 2	Panel 3	Panel 4	Panel 5	Panel 6
	(Vaccinated Sera)	(Non-vaccinated Sera)	(Hemolytic and Lipemic Sera)	(Antibody Depleted Human sera)	(High Titre Sera)	(MRS and IQC)
1.	ADK/15001	AM0001/N1/2022/IA	Hemolytic sera	Blank Human Sera	ADK/15001	S1
2.	APB/11044	DG0002/NI/2022/IA	Lipemic sera		P-V/13020	S2
3.	AVR/15027	AP0003/NI/2022/IA			SSD/11130	S3
4.	G-S/14002	SS0004/NI/2022/IA			YPI/11083	S4
5.	GSK/11123	PO0005/NI/2022/IA				S5
6.	NNF/11119	VR0006/NI/2022/IA				S6
7.	PBN/14007					S7
8.	PNI/11038					S8
9.	IQC 1					IQC 1
10.	IQC 2					IQC 1
11.	IQC 3					IQC 1
12.						IQC 2
13.						IQC 2
14.						IQC 3
15.						IQC 3

IQC, internal quality controls; MRS, multiplex reference standard; S1–S8, standard 1–8; Panel 1: samples for precision and accuracy containing High, Mid and Low level of IgG content, Panel 2–4: samples for selectivity containing negative or low IgG content; Panel 5: samples for dilution linearity containing high IgG content.; Panel 6: MRS and IQCs for solution stability.

### 2.9 Assay validation

The assay was validated based on the United States Food and Drug Administration (US FDA), European Medicines Agency (EMA), and International Council for Harmonization Multidisciplinary (ICH M10) guidelines for bioanalytical methods.

#### 2.9.1 Assay specificity

The assay specificity was demonstrated using two approaches in three different runs: (1) Approach 1 (Inhibition Approach): The MRS was incubated with each of the purified antigens (10 µg/ml of DT, TT, PT, FHA, PRN, Hib, and Hep B) for 1h, and the percentage inhibition of response was determined. Approach 2 (signal-to-noise ratio): Specificity of the assay was determined as the signal-to-noise ratio, wherein the MFI observed for assay blanks/reagents/negative human sera was compared to the MFI observed at the lowest calibrator in the reference curve. A signal-to-noise ratio of more than 5 was kept as acceptance criteria for specificity.

#### 2.9.2 Assay selectivity

The method’s selectivity was evaluated in three independent runs using three human serum matrices: (i) Matrix 1-non-vaccinated sera (Panel 2; Samples 1–6), (ii) Matrix 2- hemolytic and lipemic matrix (Panel 3; Samples 1–2), and (iii) Matrix 3-antibody depleted human sera (Panel 4; Sample 1), as mentioned in **Table 2**. These matrices are representative of negative or low-concentration sera. Matrix 1 and Matrix 2 were spiked with different concentrations of MRS and tested at 1:400 (high), 1:6,400 (medium), and 1:12,800 (low). Matrix 3 was spiked with the MRS and IQCs. Recovery of spiked samples from the different matrices was calculated with the acceptance criteria within the range of 70%–130% of expected concentrations.

#### 2.9.3 Precision

The assay precision was evaluated over three days and six runs for different analysts, days, and lots of coupled beads and PE (**Table 2**, Panel 1). Intra-assay precision refers to the variability observed for the same day. Inter-assay precision refers to the variability in experiments performed on different days by different analysts using different lots of beads and R-PE lots. The assay precision was reported as the % CV.

#### 2.9.4 Accuracy

Accuracy was assessed over three days and six runs using a panel of sera samples (**Table 2**, Panel 1). These samples were tested at different concentrations in six assays spread over three days using three different bead lots and read by two analysts. The estimates were compared to assigned unitages to determine the accuracy. The resulting IgG concentration of each serum sample was calculated and compared with the assigned values with an acceptance criterion of recovery between 70% and 130%.

#### 2.9.5 Dilution linearity

Dilution linearity was evaluated in three different runs using Panel 5 (**Table 2**). Assay dilutability was assessed in three independent runs, using twofold and fourfold dilutions starting from 1:100 till the serum sample was quantifiable. Recovery was calculated as a percentage difference between observed and assigned concentrations. Linearity is considered acceptable when said dilution complied with an acceptable % CV of duplicates (<20%) and dilution-corrected concentrations within 70%–130% of assigned values.

#### 2.9.6 Assay range

The assay range for each antigen was determined using estimates from precision, accuracy, and dilution linearity, wherein the most stringent lower and upper concentration limits complying with acceptable accuracy (75%–125%) and precision (<20% CV) and dilutional accuracy between 70% and 130% were selected. The reference standard was evaluated in six runs by twofold serial dilutions of the MRS from 1:200 to 1:25,600. The assay range was also supported by back-fitted concentrations of calibration standards. Back-fitted recoveries acceptance criterion was kept at 75%–125%.

#### 2.9.7 Limit of detection and limit of quantification

Limit of detection (LOD) and limit of quantification (LOQ) for each of the antigens were determined in three runs using curve-fitted MFI at the minimum detectable response (3× of baseline response) and minimum quantifiable response (5× of baseline response).

#### 2.9.8 Robustness

Robustness of the assay was assessed by challenging the critical assay steps with deliberate variations to set parameters. Incubation time and temperature at different steps of the assay were challenged with deliberate variations. Robustness with respect to the use of different lots of beads and secondary antibodies were evaluated. Assessment was based on the % CV difference of observed versus expected concentrations of IQCs covering the entire range of the assay.

#### 2.9.9 Solution stability

The reference standard and the IQCs from Panel 6 (**Table 2**) were evaluated for the solution stability. The solution stability of the assay was determined by analyzing the assay plates at pre-determined intervals of 0h, 12h, and 24h. Results obtained at 12h and 24h were compared with the results obtained at the initial stage (0h) to determine the hold time of the plate with the acceptance criteria of ≤20%.

### 2.10 Method comparability

Comparability of MIA (seven-plex) with commercially available kits (conventional ELISA) and other bead-based validated multiplex assays (five-plex) was carried out using international standards as assay controls. WHO international standards 06/142, 10/262, 13/240, 09/222, and 07/164, and MRS were used as assay controls for comparability assessment.

For commercial ELISA kits, assays were performed according to the manufacturer’s instructions. For IBL kits (PRN, DT, TT, and Hib), samples were diluted from 1:100 to 1:12,800 and added to pre-coated plates and incubated for 1h at 37°C, except for the Hib antigen, which was incubated at room temperature (RT) for 1h. Plates were washed three times with wash buffer, and 100 µl of enzyme conjugate (peroxidase-labeled anti-human IgG) was added to each well. This was incubated for 30 min at RT, except for the Hib antigen, which was incubated for 60 min at RT, followed by three washes. Subsequently, 100 µl of chromogen/substrate solution was added to each well and incubated for 15 min at RT and 30 min for the Hib antigen. Finally, 100 µl of stop solution was added, and the OD was measured at 450 nm using a Biotek ELISA reader (USA). The OD values within the linear part of the curve were converted to IU/ml for PRN, DT, and TT and to µg/ml for Hib antigen by interpolation from a four-parameter logistic (4-PL) standard curve.

The Euroimmun ELISA test kit was used for quantification of antibodies against PT and FHA antigens. In the first reaction step, calibrators, controls, and diluted samples were incubated in the wells, and positive samples contained specific IgG antibodies bound to the antigens. A second incubation was carried out using an enzyme-labeled anti-human IgG (enzyme conjugate), catalysing a color reaction to detect the bound antibodies. Photometric measurement of the color intensity was conducted at a wavelength of 450 nm and a reference wavelength between 620 nm and 650 nm and read within 30 min of the stop solution being added.

The Bio-Rad ELISA test kit was used for the quantification of human antibodies against the Hep B IgG class in 09/222, 07/164, and MRS. In the first reaction step, calibrator, controls, and diluted samples were incubated in the wells; samples contained specific IgG antibodies bound to the antigens coated on the plate. A second incubation was carried out using an enzyme-labeled anti-human IgG (enzyme conjugate), catalyzing a color reaction to detect the bound antibodies. Photometric measurement of the color intensity was made at a wavelength of 450 nm and a reference wavelength between 615 nm and 630 nm and read within 30 min of adding the stop solution. The results of this assay were compared with the bead-based assay.

A validated five-plex bead-based multiplex assay (16) for DT, TT, PT, FHA, and PRN was also used for comparability. Unitages of five-plex MRS (which is a mix of 06/142, 10/262, and TE-3) for PT, FHA, PRN, DT, and TT antigens were verified at the Medicines and Healthcare Products Regulatory Agency (MHRA) laboratory using conventional ELISA assays.

#### 2.10.1 Statistical analysis

Calibration curves were generated using the 5-PL logistic fit, and the values for back-fitted recoveries were set between 75% and 125%, and the % CV values were at ≤ 20%. Statistical analyses were performed using Microsoft Office Excel 2019 and statistical software GraphPad Prism 7.05. The following formula was used for method validation parameters:

The percentage recovery for selectivity assessment was calculated as follows:


$$\begin{array}{c} (Observed\;concentration\;of\;spiked\;sample\\ -Observed\;concentration\;of\;unspiked\;sample)\\ \;÷Expected\;concentartion\times 100 \end{array}$$

The following equations were used to calculate MFI for the determination of LOD and LOQ:

Minimum Detectable Response = Avg. blank human serum MFI+(3 * SD of blank serum MFI)
Minimum Quantifiable response = 5* (MFI at minimum detectable response)

The lowest quantifiable response was multiplied with 200 (minimum two sera dilutions, i.e., 100 and 200) to obtain LOQ in IU/ml and µg/ml.

## 3 Results

### 3.1 Optimization of bead coupling procedures

**Figure 2:**
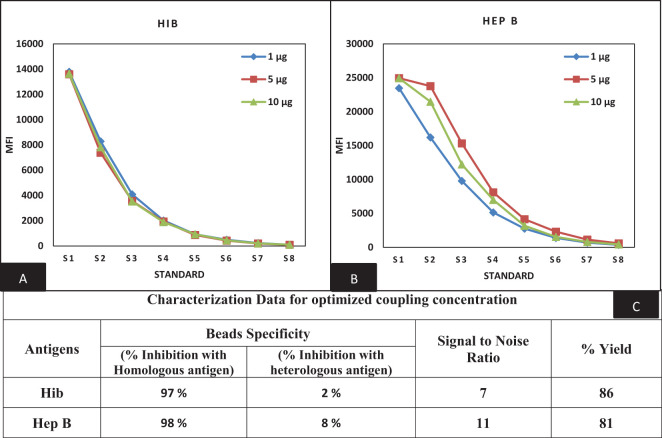
Optimization of bead coupling procedures **(A, B)** represents dilution versus MFI graphs at three different coupling concentrations for Hib and Hep B antigens. The concentration of 1 µg (Hib) and 5 µg (Hep B) were found optimum for the coupling. **(C)** represents the characterization data of bead coupling at 1 µg and 5 µg with respect to suitability parameters of bead specificity, signal to noise ratios and yields comparison. Hib, Haemophilus influenza b; Hep B, Hepatitis B.

The beads provided by Luminex of predetermined spectral regions were used for the conjugation of targeted proteins. We previously reported the conjugation procedure for PT, FHA, PRN, DT, and TT antigens (16). A similar procedure of the EDAC/Sulfo-NHS was followed for coupling optimization of Hib and Hep B antigens. Varying concentrations of Hib and Hep B antigens (1, 5, and 10 µg) were evaluated for the selection of optimal coating concentration. Concentrations of 1 and 5 µg of Hib and Hep B antigens were found to be optimal as determined by the dose-response curve (**Figure 2**). Coupled beads generated dose-response curves with a stable assay range with backfitted recoveries of 75%–125% and comparable MFIs between mono- and multiplex conditions using human sera. MFIs were comparable (≤20% CV), and differences were observed supporting the suitability for use in MIA. Homologous and heterologous inhibitions were noted at 85% and 15% at the optimal coupling concentration and thereby supporting the suitability of the coupling method (**Figure 2**).

### 3.2 Characterization and suitability of international reference standards

As a first step of characterization, the available reference standards were screened to assess the presence of antibodies against seven target antigens. **Table 3** provides the results of the screening study. It was noted that among all the international reference standards, except for 06/142, all the other standards showed the presence of antibodies against all seven antigens. 06/142 was positive for six antigens and was found to be below LOQ for Hepatitis B IgG antibodies. 09/222 was found to have the maximum number of antibodies against Hib (74.04 µg/ml) antigen, as expected. Whereas 07/164, showed the highest number of antibodies against PT (101.24 IU/ml), FHA (154.47 IU/ml), PRN (167.93 IU/ml), DT (2.11 IU/ml), TT (34.31 IU/ml), and Hep B (102.75 IU/ml) antigens. Based on the screening study, an equi-mix of 09/222 and 07/164 was selected to be developed as MRS for the seven-plex assay, as it will allow maximum assay range for all seven antigens.

**Table 3:** Characterization of WHO reference standards for development of MRS.

WHO reference standard	IU/ml						µg/ml
	PT	FHA	PRN	DT	TT	Hep B	Hib
06/142	**92.96** **(88%)**	**115.90** **(95%)**	**40.61** **(104%)**	0.19	2.02	ND	0.56
10/262	86.72	119.21	146.67	**1.90** **(95%)**	11.46	2.61	4.34
13/240	153.25	262.64	202.13	6.55	**40.55** **(90%)**	3.59	10.28
09/222	14.46	29.95	31.78	0.48	15.50	9.18	**74.04** **(107%)**
07/164	101.24	154.47	167.93	2.11	34.31	**102.75** **(103%)**	4.60

PT, pertussis toxin; FHA, filamentous hemagglutinin; PRN, pertactin; DT, diphtheria toxoid; TT, tetanus toxoid; Hep B, hepatitis B; Hib, Haemophilus influenzae b; IU/ml, International units per milliliter; µg/ml, microgram per milliliter; WHO, World Health Organization; ND, not detected.

WHO reference standards were screened for IgG antibodies against PT, FHA, PRN DT, TT, Hib, and Hep B using a bead-based assay. Values in bold indicate the observed concentration, and values in parenthesis indicate percentage agreement with official unitages.

### 3.3 Assignment of MRS unitages and verification

The unitages of MRS (equi-mix of 09/222 and 07164) for DT, TT, PT, FHA, PRN, Hep B, and Hib IgG antibodies were assigned using data from six independent runs (**Table 4**). The assigned unitages were verified using international reference standards of respective antigens as controls. Verification studies suggest concordance (values obtained by the seven-plex assay were within 80%–120% of expected values) for all the international standards. The values obtained by MIA for international standards were also compared with estimates from commercially available diagnostic kits and a validated bead-based five-plex assay reported previously (16), and results suggest excellent agreement (80%–120%) with the assigned values (**Table 5**).

**Table 4:** Assigned unitages of multiplex reference standard (MRS).

Antigen	Assigned unitages	% CV
PT (IU/ml)	57.85	**16**
FHA (IU/ml)	92.21	**9**
PRN (IU/ml)	99.86	**9**
DT (IU/ml)	1.29	**11**
TT (IU/ml)	24.90	**7**
Hep B (IU/ml)	55.96	**3**
Hib (µg/ml)	39.32	**9**

PT, pertussis toxin; FHA, filamentous hemagglutinin; PRN, pertactin; DT, diphtheria toxoid; TT, Tetanus toxoid; Hep B, Hepatitis B; Hib, Haemophilus influenzae b; IU/ml, International units per milliliter; µg/ml, microgram per milliliter; % CV, percentage coefficient of variation.

Multiplex reference standard is an equimolar mixture of 09/222 and 07/164. Based on the characterization study, unitages were assigned to MRS. Values are representation of mean (*N* = 6 assays). Values in bold indicate the % CV of 6 assays.

**Table 5:** Verification of MRS unitages using international reference standards. .

Antigen	Reference standards batch no.	7-plex assay	Commercial kit	Pentaplex assay	% CV
PT (IU/ml)	06/142 (aP)	**113 (107%)**	**115 (108%)**	**95 (90%)**	10
	10/262 (DT)	83	61	64	17
	13/240 (TT)	116	104	126	10
	09/222 (Hib)	17	21	NA	15
	07/164 (Hep B)	111	107		3
	MRS	63	59		5
FHA (IU/ml)	06/142 (aP)	**126 (103%)**	**108 (89%)**	**112 (92%)**	8
	10/262 (DT)	111	106	107	2
	13/240 (TT)	261	272	219	11
	09/222 (Hib)	35	32	NA	6
	07/164 (Hep B)	153	147		3
	MRS	92	86		5
PRN (IU/ml)	06/142 (aP)	**41 (105%)**	**43 (110%)**	**35 (90%)**	10
	10/262 (DT)	163	146	132	11
	13/240 (TT)	216	193	158	15
	09/222 (Hib)	31	28	NA	7
	07/164 (Hep B)	174	162		5
	MRS	107	102		3
DT (IU/ml)	06/142 (aP)	0.2	0.2	0.2	0
	10/262 (DT)	**1.9 (95%)**	**1.7 (85%)**	**1.8 (90%)**	6
	13/240 (TT)	6.9	6	5.4	12
	09/222 (Hib)	0.5	0.4	NA	16
	07/164 (Hep B)	2	1.9		4
	MRS	1.2	1.3		6
TT (IU/ml)	06/142 (aP)	2	2	2	0
	10/262 (DT)	11	14	11	14
	13/240 (TT)	**42 (93%)**	**44 (98%)**	**41 (91%)**	10
	09/222 (Hib)	16	19	NA	12
	07/164 (Hep B)	35	31		9
	MRS	27	29		5
Hib (µg/ml)	09/222 (Hib)	**73 (105%)**	**74 (107%)**	NA	1
	07/164 (Hep B)	2	2.4		13
	MRS	44	37		12
Hep B (IU/ml)	09/222 (Hib)	10	8	NA	16
	07/164 (Hep B)	**110 (110%)**	**98 (98%)**		8
	MRS	62	54		10

PT, pertussis toxin; FHA, filamentous hemagglutinin; PRN, pertactin; DT, diphtheria toxoid; TT, tetanus toxoid; Hib, Haemophilus influenzae b; Hep B, hepatitis B; IU/ml, international units per milliliter; µg/ml, microgram per milliliter; MRS, multiplex reference standard; NA, not applicable; % CV, percentage coefficient of variation. Values in bold indicate the observed concentration, and values in parenthesis indicate percentage agreement with official unitages. % CV value represents the variations between seven-plex bead-based assay (*N* = 6), commercial ELISA (*N* = 3) and pentaplex assay (*N* = 12).

### 3.4 Reference standard curve for MIA

**Figure 3:**
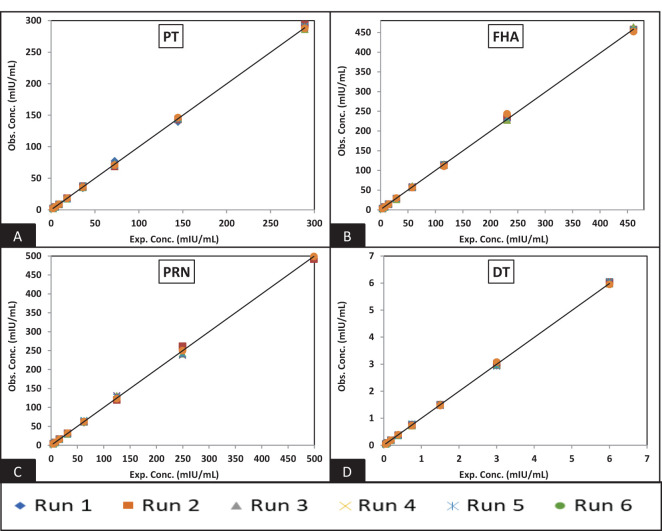
Dynamic range of MRS: **(A–D)** represent the assay range of MRS for PT, FHA, PRN, and DT antigens. The x-axis represents expected concentration (mIU/ml), whereas the Y-axis represents obtained concentration (mIU/ml). The data is representative of six runs. PT, pertussis toxin; FHA, filamentous haemagglutinin; PRN, pertactin; DT, diphtheria toxoid; milli-international units per milliliter.

**Figure 4:**
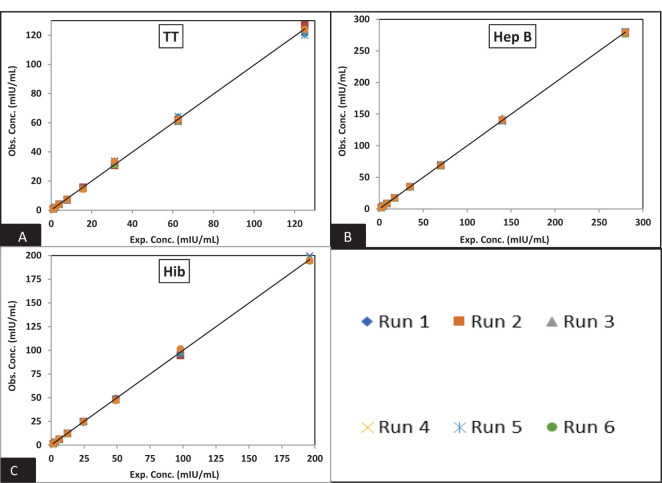
Dynamic range of MRS: **(A–C)** represents the assay range of MRS for TT, Hib, and Hep B antigens. The x-axis represents expected concentration (mIU/ml) whereas the y-axis represents obtained concentration (mIU/ml). Data are representative of six runs. TT, tetanus toxoid; Hib, Haemophilus influenzae b; Hep B, hepatitis B; mIU/ml, milli-international units per milliliter.

**Figures 3** and **4** depict the validated reference standard curve range for all seven antigens. The estimates from accuracy, precision, and robustness experiments were also considered to support the upper and lower limits of the assay range. **Table 6** provides the upper limit (UL) and lower limit (LL) of quantifications, as observed in different validation parameters, which ranged from 2.26–289 mIU/ml for PT, 3.60–461 mIU/ml for FHA, 3.90–499 mIU/ml for PRN, 0.05–6 mIU/ml for DT, 0.98–125 mIU/ml for TT, 1.53–196 ng/ml for Hib, and 2.19–280 mIU/ml for Hep B (**Table 6**). 5-PL back-fitted recoveries were used to establish the linear range of the assay. The LL and UL of the assay range were determined using estimates from accuracy, precision, and dilutional linearity (**Table 6**).

**Table 6:** Final assay range with a lower and upper limit of quantification.

Antigen	Precision		Accuracy		Dilutional linearity of sample		Dilutional linearity of standard		Calibration curve range	
	Lower limit	Upper limit	Lower limit	Upper limit	Lower limit	Upper limit	Lower limit	Upper limit	Lower limit	Upper limit
PT (mIU/ml)	10.54	168.70	10.54	168.70	**2.26**	289	0.14	289	2.26	289
FHA (mIU/ml)	17.08	273.30	17.08	273.30	**3.6**	461	0.23	461	3.6	461
PRN (mIU/ml)	13.72	219.47	13.72	219.47	**3.9**	499	0.24	499	3.9	499
DT (mIU/ml)	0.32	5.18	0.32	5.18	**0.05**	6	0.003	6	0.05	6
TT (mIU/ml)	3.21	51.40	3.21	51.40	**0.98**	125	0.06	125	0.98	125
Hib (ng/ml)	3.06	52.06	3.06	52.06	**1.53**	196	1.53	196	1.53	196
Hep B (mIU/ml)	4.16	66.57	4.16	66.57	**2.19**	280	0.14	280	2.19	280

PT, pertussis toxin; FHA, filamentous hemagglutinin; PRN, pertactin; DT, diphtheria toxoid; TT, tetanus toxoid; Hep B, hepatitis B; Hib, Haemophilus influenzae b; mIU/ml, milli-international unit per milliliter; ng/ml, nanogram per milliliter.

Data is representative of estimates in precision, accuracy, and dilution linearity validation parameters. Precision, accuracy, and dilution linearity estimates to support calibration curve range. Values highlighted in bold were used to estimate the lower limit.

### 3.5 Assay validation

#### 3.5.1 Assay specificity

Specificity was demonstrated using two approaches: (a) inhibition experiments and (b) signal-to-noise ratio. **Table 7** demonstrates the data for inhibition experiments wherein the addition of homologous antigen demonstrated more than 90% inhibition, whereas heterologous inhibition was found below 20% indicating high specificity of the assay. This is further supported by data from assay buffers and negative sera samples wherein a signal-to-noise ratio was >20 for all antigens. (MFIs of assay buffers/negative sera samples) (**Table 7**).

**Table 7:** Specificity of assay.

Inhibitor		Approach 1 (% Inhibition)								
		PT	FHA	PRN		DT	TT		Hib	Hep B
PT		**97**	19	10		5	0		15	1
FHA		13	**93**	13		1	1		4	0
PRN		7	1	**98**		5	1		13	1
DT		9	0	0		**98**	1		8	0
DT		4	1	6		0	**95**		-1	0
Hib		-2	1	1		-3	2		**97**	0
Hep B		-1	0	-1		-6	0		8	**98**
Approach 2 (signal-to-noise ratio)										
Antigens	MFI of lowest standard in calibration curve				Signal-to-noise ratio using negative sera sample			Signal-to-noise ratio using assay buffer		
PT	210				210			208		
FHA	371.8				372			247		
PRN	110.8				110			110		
DT	329.3				165			180		
TT	2621.8				131			174		
Hib	22.5				22			20		
Hep B	395.3				303			312		

PT, pertussis toxin; FHA, filamentous hemagglutinin; PRN, pertactin; DT, diphtheria toxoid; TT, tetanus toxoid; Hep B, hepatitis B; Hib, Haemophilus influenzae B; MFI, median fluorescence intensity.

**Table 7** represents the specificity study approach 1 and approach 2. Approach 1 data indicates the percentage inhibition on addition of homologous and heterologous inhibitor. Values in bold indicates the % inhibition in MFI response of reference standard in case of homologous condition when individual antigens were added at the concentration of 10 µg/ml.

Approach 2 data represents the signal to noise ratio of an assay in presence of blank sera and assay buffer (Luminex Assay Buffer) indicating no cross talk from the assay reagents.

#### 3.5.2 Assay selectivity

The selectivity of the method was evaluated with respect to the use of different serum matrices covering hemolytic, lipemic, non-vaccinated, and antibody-depleted sera. The assay was found to be highly selective, wherein spike recoveries (80%–120%) were observed in all the matrices (**Table 8**). No interference was observed in the assay for hemolytic and lipemic matrices covering up to 2.02 g/dl of hemoglobin and 275 mg/dl of total cholesterol, respectively.

**Table 8:** Selectivity assessment in different matrices.

Matrix	Samples	MRS spike level	Spike % recovery						
			PT	FHA	PRN	DT	TT	Hib	Hep B
Matrix 1 (Negative sera samples)	Sample 1	High	100	105	94	90	98	109	95
		Middle	92	93	91	81	97	101	93
		Low	97	110	112	100	110	101	110
	Sample 2	High	97	100	96	88	99	102	91
		Middle	90	93	94	88	102	106	99
		Low	110	111	107	100	109	104	103
	Sample 3	High	93	93	90	84	93	101	88
		Middle	96	98	96	89	102	109	91
		Low	99	98	102	93	100	97	104
	Sample 4	High	97	98	92	87	98	106	93
		Middle	97	97	97	90	98	102	99
		Low	102	107	102	93	107	105	101
	Sample 5	High	85	87	87	80	92	91	88
		Middle	84	87	92	83	91	96	87
		Low	97	91	94	90	98	97	95
	Sample 6	High	86	87	86	83	94	89	87
		Middle	83	85	88	82	88	93	86
		Low	110	98	100	93	102	91	106
Matrix 2 (hemolytic and lipemic samples)	Sample 1	High	92	86	90	83	97	96	89
		Middle	95	97	95	90	98	103	95
		Low	114	112	110	113	118	119	119
	Sample 2	High	92	91	94	86	99	95	91
		Middle	105	102	95	99	106	112	104
		Low	117	119	113	117	120	118	119
Matrix 3 (antibody depleted human serum)	Sample 1	IQC 1	95	94	106	103	113	86	95
		IQC 2	89	89	96	94	90	81	85
		IQC 3	87	86	94	99	90	88	81

PT, pertussis toxin; FHA, filamentous hemagglutinin; PRN, pertactin; DT, diphtheria toxoid; TT, tetanus toxoid; Hep B, hepatitis B; Hib, Haemophilus influenzae B; MRS, multiplex reference standard.

IQC: internal quality control; Matrix 1: panel for selectivity parameters containing non-vaccinated sera samples; Matrix 2: hemolytic and lipemic sera; Matrix 3: Antibody depleted human sera.

Selectivity was assessed using spike recovery experiments in different serum matrices.

#### 3.5.3 Precision

Precision analysis suggested that the assay was precise for different analysts, on different days, using different lots of beads and PE. % CV for the combined precision of the two analysts was below 20% for all the antigens (**Table 9**). Based on the data, LL and UL based on precision ranged from 10.54–168.70 mIU/ml for PT, 17.08–273.30 mIU/ml for FHA, 13.72–219.47 mIU/ml for PRN, 0.32–5.18 mIU/ml for DT, 3.21–51.40 mIU/ml for TT, 3.06–52.06 ng/ml for Hib and 4.16–66.57 for Hep B (**Table 6**).

**Table 9:** Precision and accuracy estimates.

Samples	Precision																				
	*Analyst (% CV)							**Days (% CV)							***Bead Lot (% CV)						
	PT	FHA	PRN	DT	TT	Hep B	Hib	PT	FHA	PRN	DT	TT	Hep B	Hib	PT	FHA	PRN	DT	TT	Hep B	Hib
Sample 1	6	9	12	13	10	14	1	20	13	18	16	14	15	15	16	8	17	12	7	10	9
Sample 2	6	4	5	5	5	9	14	12	8	8	11	7	11	12	13	7	10	7	7	8	7
Sample 3	10	5	9	13	12	11	9	15	14	13	16	17	15	16	13	10	12	11	15	10	11
Sample 4	13	11	12	19	8	14	9	20	10	11	16	11	12	17	12	8	11	12	14	12	20
Sample 5	6	5	6	5	3	4	15	12	6	6	6	6	7	11	9	6	5	4	8	7	10
Sample 6	5	4	7	7	5	5	4	10	8	6	7	7	8	19	8	6	4	4	8	7	12
Sample 7	9	7	13	8	12	8	12	13	15	13	10	13	11	15	11	15	7	9	8	9	14
Sample 8	12	2	4	2	5	2	12	14	15	12	12	12	11	12	5	10	6	6	9	7	8
Samples	Accuracy																				
	*Analyst (% Recovery)							**Days (% Recovery)							***Bead Lot (% Recovery)						
	PT	FHA	PRN	DT	TT	Hep B	Hib	PT	FHA	PRN	DT	TT	Hep B	Hib	PT	FHA	PRN	DT	TT	Hep B	Hib
Sample 1	81	93	95	82	93	79	80	89	94	98	87	95	82	85	94	94	89	85	93	80	89
Sample 2	86	90	86	84	89	79	85	101	95	94	99	94	88	84	102	93	94	90	92	88	90
Sample 3	84	84	83	86	93	89	84	95	87	86	89	99	91	89	98	90	86	89	103	91	96
Sample 4	80	95	98	79	104	86	104	91	95	101	90	107	89	118	96	97	102	96	107	93	112
Sample 5	89	97	95	93	101	92	92	92	93	96	92	100	91	93	92	93	92	90	98	88	99
Sample 6	100	109	104	101	112	111	97	102	102	104	101	110	106	96	104	102	102	101	110	103	101
Sample 7	82	117	112	107	121	104	93	90	105	105	104	115	101	89	93	102	101	102	112	98	96
Sample 8	95	103	103	99	117	96	93	102	102	103	97	116	95		105	98	101	95	110	93	101

PT, pertussis toxin; FHA, filamentous hemagglutinin; PRN, pertactin; DT, diphtheria toxoid; TT, tetanus toxoid; Hep B, hepatitis B; Hib, Haemophilus influenzae b; % CV, percentage coefficient of variation.

Samples 1–8 represent the panel 1 containing vaccinated sera samples. Precision and Accuracy results are determined concerning different analysts, days, and bead lots. Precision is determined in terms of % CV. Accuracy is reported in terms of % recovery. *Combined precision (% CV) and accuracy (% recovery) of analysts’ 1 and 2, **Combined precision and accuracy of 6 runs over 3 days, ***Combined precision and accuracy of multiple bead lots.

#### 3.5.4 Accuracy

Acceptable recoveries were observed within the range of 80%–120% for PT, FHA, PRN, DT, TT, and Hep B antigens (**Table 9**). The LL and ULs based on accuracy ranged from 10.54 to 168.70 mIU/ml for PT, 17.08 to 273.30 mIU/ml for FHA, 13.72 to 219.47 mIU/ml for PRN, 0.32 to 5.18 mIU/ml for DT, 3.21 to 51.40 mIU/ml for TT, 3.06 to 52.06 ng/ml for Hib, and 4.16 to 66.57 for Hep B (**Table 6**).

#### 3.5.5 Dilution linearity

**Figure 5:**
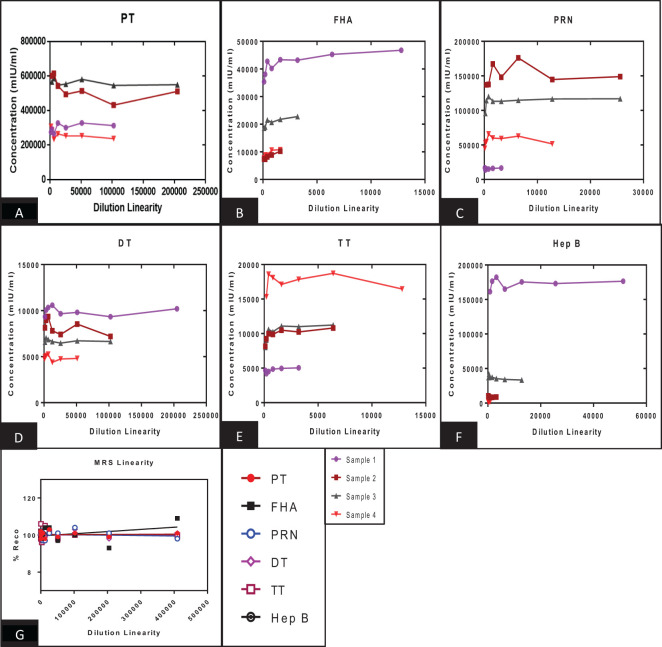
Dilution linearity of assay in high titre sera samples and MRS for PT, FHA, PRN, DT, TT, and Hep B antigens. **(A–F)** represents the dilution linearity of four samples for PT, FHA, PRN, DT, TT, and Hep B antigens. The x-axis represents the sample’s dilutions, and the y-axis represents the concentration observed in mIU/ml. **(G)** represents the dilution linearity data for MRS. Sera samples and MRS shows no loss of dilution integrity over the dilution range. PT, pertussis toxin; FHA, filamentous haemagglutinin; PRN, pertactin; DT, diphtheria toxoid; TT, tetanus toxoid; Hep B, hepatitis B; MRS, multiplex reference standard.

The panel samples were tested in three independent runs across a series of sera samples ranging from a dilution of 1:100 to 1:409,600. No loss in dilution integrity was observed, with a twofold increase in dilution ranges up to 1:409,600 dilution fold studied for the respective antigens (**Figure 5**).

#### 3.5.6 Assay range

The assay range was selected based on the estimates from precision, accuracy, and dilutional linearity study sets. LL and UL of assay range were established ranging from 2.26 to 289 mIU/ml for PT, 3.60 to 461 mIU/ml for FHA, 3.90 to 499 mIU/ml for PRN, 0.05 to 6 mIU/ml for DT, 0.98 to 125 mIU/ml for TT, 1.53 to 196 ng/ml for Hib, and 2.19 to 280 mIU/ml for Hep B. The LL of an assay range was the lowest concentration that showed acceptable precision, accuracy, and dilution linearity experiments (**Table 6**).

#### 3.5.7 Robustness

**Figure 6:**
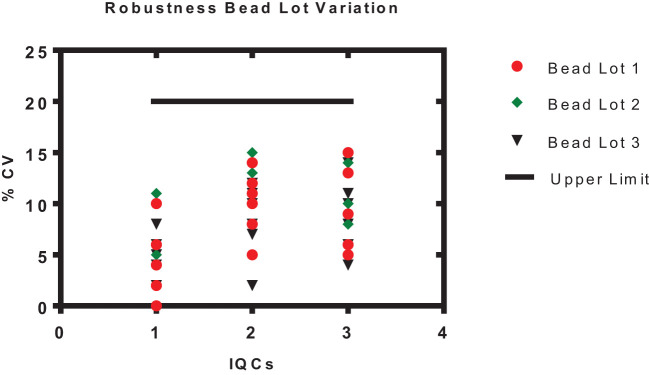
Robustness of an assay with different bead lots. The x-axis represents the samples 1–3 (IQC1-IQC3), and the y-axis represents the % CV of the observed concentration compared with the expected concentration of an IQCs. Each data point represents the antigens PT, FHA, PRN, DT, TT, and Hep B.

The robustness of the assay was studied using IQCs covering the entire assay range. The critical assay parameters studied included incubation time with beads, incubation time with PE, incubation temperature of beads and PE, different lots of PE, and different bead lots (**Figure 6**). % CV of observed versus expected concentrations was calculated for each IQC. The results demonstrated that concentrations of IQCs generated from the assays with deliberate variations were within the acceptable range of<20% variability for all the antigens (**Table 10**).

**Table 10:** Assay robustness.

% CV												
Antigen	IQC	Sample incubation time		R-PE incubation time		Incubation temperature		Different R-PE lots		Solution stability		
		50 min	70 min	20 min	40 min	32°C	42°C	PE Lot 1	PE Lot 2	0 hr	12 hr	24 hr
PT	IQC 1	14	12	6	4	6	4	14	4	6	3	5
	IQC 2	19	17	14	9	8	10	16	6	19	9	6
	IQC 3	15	11	17	3	8	8	14	3	15	13	7
FHA	IQC 1	13	11	4	5	5	0	13	4	5	5	3
	IQC 2	18	12	6	13	7	6	17	8	18	8	22
	IQC 3	15	10	7	12	4	4	13	0	11	11	27
PRN	IQC 1	4	2	1	1	1	6	5	3	5	1	6
	IQC 2	7	8	6	7	2	1	4	1	5	0	24
	IQC 3	3	2	8	2	5	6	4	8	6	3	32
DT	IQC 1	7	6	6	3	4	4	11	0	3	4	4
	IQC 2	12	13	11	12	7	8	12	1	8	1	3
	IQC 3	7	2	10	5	3	1	7	8	3	1	4
TT	IQC 1	5	2	2	5	2	11	6	6	0	0	2
	IQC 2	13	12	11	12	2	3	10	2	12	6	2
	IQC 3	9	2	9	6	3	6	8	6	10	9	1
Hib	IQC 1	8	13	10	10	3	7	11	6	14	14	12
	IQC 2	13	18	13	15	4	7	11	4	11	10	9
	IQC 3	9	12	10	10	8	13	6	3	15	12	6
Hep B	IQC 1	9	8	4	2	1	5	4	1	4	2	0
	IQC 2	13	14	8	7	1	0	10	3	15	9	5
	IQC 3	16	7	10	2	5	5	7	1	15	12	0

PT, pertussis toxin; FHA, filamentous hemagglutinin; PRN, pertactin; DT, diphtheria toxoid; TT, tetanus toxoid; Hep B, hepatitis B; Hib, Haemophilus influenzae b; % CV, percentage coefficient of variation; IQC, internal quality control; R-PE, Phycoerythrin; The data represents the % CV observed for deliberate variations in critical assay parameters for all the seven antigens. % CV represents the percent difference between assigned and values observed post deliberate variation in parameter.

#### 3.5.8 Solution stability

The results of solution stability at predetermined intervals of 0h, 12h, and 24h suggest that a plate hold time exceeding 12h would not be suitable for routine analysis, as we observed an impact on FHA and PRN antigens after 12h of plate hold time.

### 3.6 Comparability of MIA with commercially available kits and other multiplex assays

The international reference standards as controls were analyzed using MIA, conventional ELISA, and other validated multiplex assays (**Table 5**). The unitages obtained for international standards were found to be within 80%–120% agreement with their designated unitages, supporting the verification of MRS unitages and also the comparability of MIA with conventional ELISA and multiplex assay.

## 4 Discussion and conclusion

The quantification of antigen-specific antibodies in combination vaccines is crucial for evaluating immune responses and thereby vaccine efficacy. Conventional plate-based ELISA methods are largely used but have several limitations, such as high reagent requirements, less throughput, time and labour intensive, and, most importantly, they need large sera quantities, which becomes challenging in paediatric trials. Recent advancements in MIAs, such as Luminex xMAP and Meso Scale Discovery (MSD) platforms, provide a more efficient alternative. Although these technologies may incur initial setup costs, long-term advantages in higher sensitivity, high throughput, and reduced sera volumes make them cost-effective solutions. The use of multiplex assays in serology has expanded widely, especially during the COVID-19 pandemic, where over 85 assays gained FDA authorization. Beyond SARS-CoV-2, multiplex serology has also been applied successfully for the simultaneous detection of antibodies to pneumococcal serotypes, malaria, and human papillomavirus (HPV). Previously, we reported development and validation of a five-plex Luminex serological assay to simultaneously measure IgG antibodies against TT, DT, PT, FHA, and PRN antigens (16). There are limited or no reports on multiplex assays that can simultaneously estimate Hib and Hep B antigens along with TT, DT, or aP antigens. Traditionally, the quantification of IgG concentrations against both Hib and Hepatitis B has been performed using commercial ELISA kits (17, 18).

To develop a bead-based MIA, as a first step, it is important to optimize the coupling conditions for the antigen. We have previously described the coupling procedures for T, D, and aP antigens using magnetic beads for protein-based antigens. Similar procedures were used for the Hep B antigens, which resulted in high coupling efficiency, specificity, and selectivity. However, the optimization of Hib coupling to beads is challenging. These challenges were expected due to the polysaccharide nature of the antigen. EDAC-based coupling chemistry using a specific size range of the Hib polysaccharide yielded optimum results. Additionally, a standardized reagent for Hib ELISA for capturing IgG antibodies is available at the NIBSC. This reagent is HSA-conjugated PRP and is used for coupling experiments. NIBSC reagent and EDAC-coupled Hib antigen demonstrated comparable specificity (97% inhibition with homologous antigen) and yield.

Robust assay validation relies on the availability of international standards. Monoplex international reference standards are available from NIBSC for all antigens in the panel. However, their suitability for multiplex assays needs to be assessed. All five NIBSC reference standards 06/142 (AP antigens: PT, FHA, and PRN), 10/262 (DT), 13/240 (TT), 09/222 (Hib), and 07/164 (Hep B) were studied for suitability. An equi-mix of reference standards 07/164 and 09/222 (MRS) was proposed as a suitable reference standard for the seven-plex assay, as 07/164 reported the highest amount of antibodies against PT, FHA, PRN, DT, Hep B, and TT, whereas 09/222 was found to have the highest amount of antibodies against Hib antigen alone (**Table 3**). The unitages of MRS were further verified by cross-calibration, wherein the equi-mix reference standard generated accurate unitages of other international reference standards, such as those of pertussis, tetanus, and diphtheria antigens. The validation of these units holds significant potential, paving the way for comparative inter-laboratory studies for multiplex assays.

The multiplex assay requires the development of a common standard curve that can accurately quantify each antigen within a shared dilution series. However, accommodating all antigens within a common dilution range imposes constraints on the dynamic range of individual antigens. As a result, certain antigens may exhibit enhanced sensitivity, while others may experience a slight reduction in their measurable range. In this study, compared to our previous five-plex assay, a slight loss in sensitivity for PT, FHA, and TT was observed. While DT showed improvement in sensitivity, PRN was not impacted at all. This loss in sensitivity was not found to be significant, as assay LOQs are still 30- to 40-fold higher than those of commercially available assays. The LOQ of Hepatitis B antibodies was found to be three to fivefold higher than commercially available assays such as kits from Bio-Rad, Abbott. The method met the FDA, EMA, and ICH M10 guidance requirements for assay range, selectivity, specificity, precision, accuracy, linearity, LOD, LOQ, and solution stability. The assay demonstrated robustness against variations in incubation time, temperature, and different lots of reagents. Commercially available IgG ELISA kits are widely used to assess antibody responses to aP-based combination vaccines. We also compared the results obtained from the MIA with those of commercially available ELISA kits calibrated against international reference standards. Strong concordance was observed between MIA and commercial ELISA kits for aP, DT, TT, Hib, and Hep B antigens. The method is currently being used for an approved clinical study for licensure of the DTaP-IPV-Hib + Hep B combination vaccine in India (CTRI/2020/09/027552).

Coupling of polysaccharide-based antigens to beads is challenging. There are very few publications on polysaccharides such as Hib (28). In our study, Hib antigen-coupled beads displayed consistent capture of IgG with an LOQ of 0.612 µg/ml using the NIBSC reference standard. An LOQ of 0.612 µg/ml has further scope for improvement, as commercially available assays have reported an LOQ of 0.1 µg/ml. Experiments to further increase the sensitivity of the Hib antigen are in progress, including changes in the coupling conditions or the use of other conjugated antigens. It is further noted that the Hib antigen did not show any challenges concerning any other validation parameters.

In conclusion, our study provides a validated multiplex assay for the simultaneous estimation of IgG concentrations in sera using WHO international standards. Furthermore, since the assay is developed using Luminex technology, it provides opportunities for further expansion to include new antigens in the future.

## Data Availability

The original contributions presented in the study are included in the article/Supplementary Material. Further inquiries can be directed to the corresponding author.
